# Clinical features and risk factors analysis of bronchitis obliterans due to refractory *Mycoplasma pneumoniae* pneumonia in children: a nomogram prediction model

**DOI:** 10.1186/s12879-021-06783-4

**Published:** 2021-10-21

**Authors:** Qi Cheng, Han Zhang, Yunxiao Shang, Yuetong Zhao, Ye Zhang, Donglin Zhuang, Xuxu Cai, Ning Chen

**Affiliations:** Department of Pediatrics, Shengjing Hospital of China Medical University, No. 36 Sanhao Street of Heping District, Shenyang, China

**Keywords:** Refractory *Mycoplasma pneumoniae* pneumonia, Bronchitis obliterans, Fiberoptic bronchoscopy, Nomogram model, Prediction

## Abstract

**Background:**

Early prediction of bronchitis obliterans (BO) is of great significance to the improvement of the long-term prognosis of children caused by refractory *Mycoplasma pneumoniae* pneumonia (RMPP). This study aimed to establish a nomogram model to predict the risk of BO in children due to RMPP.

**Methods:**

A retrospective observation was conducted to study the clinical data of children with RMPP (1–14 years old) during acute infection. According to whether there is BO observed in the bronchoscope, children were divided into BO and the non-BO groups. The multivariate logistic regression model was used to construct the nomogram model.

**Results:**

One hundred and forty-one children with RMPP were finally included, of which 65 (46.0%) children with RMPP were complicated by BO. According to the multivariate logistic regression analysis, WBC count, ALB level, consolidation range exceeding 2/3 of lung lobes, timing of macrolides, glucocorticoids or fiber bronchoscopy and plastic bronchitis were independent influencing factors for the occurrence of BO and were incorporated into the nomogram. The area under the receiver operating characteristic curve (AUC-ROC) value of nomogram was 0.899 (95% confidence interval [CI] 0.848–0.950). The Hosmer–Lemeshow test showed good calibration of the nomogram (p = 0.692).

**Conclusion:**

A nomogram model found by seven risk factor was successfully constructed and can use to early prediction of children with BO due to RMPP.

## Background

Mycoplasma pneumonia (MP) is one of the common pathogens that cause community-acquired pneumonia (CAP) in children and adolescents [1, 2]. Previously, *Mycoplasma pneumoniae* pneumonia (MPP) was considered as a benign self-limiting disease [3]. However, it has been found in recent years that some cases who have been treated with macrolide antibiotics for 7 days or more, still show aggravation of clinical signs, persistent fever, and aggravation of lung imaging, which is defined as refractory MPP (RMPP) [4]. In recent years, the incidence of RMPP complications is relatively high, even including necrotizing pneumonia (NP), bronchitis obliterans (BO) or thrombosis [5, 6].

Bronchitis obliterans is a chronic obstructive airflow syndrome associated with fibrous tissue hyperplasia. Its pathological features are inflammatory granulation tissue, fibrosis, small and medium airway occlusion and bronchiectasis occlusion [7]. Due to its poor prognosis and lack of effective treatments, the identification of predictors of BO has become one of the hotspots of clinical research. The prognosis of BO to a large extent depends on the timing of diagnosis and intervention [8]. Therefore, it is quite essential to find a feasible and accurate screening tool to identify the BO high-risk population of RMPP patients, which will help the early intervention of BO.

The nomogram is a popular prognostic tool that can predict clinical events by integrating potential risk factors [9]. The nomogram has been widely used in tumor prognosis and supports the development of personalized oncology medicine [10]. Recently, a nomogram has been effectively used to predict the short-term and long-term survival rates of asymptomatic adults screened for cardiac risk factors [11]. Moreover, a nomogram is also used to predict the risk stratification of diseases, including myocardial infarction and stroke [12, 13]. However, no previous studies have reported relevant studies on the risk prediction of BO in RMPP patients with nomograms. In the current study, we retrospectively analyzed the clinical characteristics, risk factors, and the application and timing of corresponding treatment measures in children with RMPP and BO. Importantly, we established a nomogram prediction model to predict the risk of BO in children with RMPP, with the hope to provide ideas for the early diagnosis of BO and the timing of intervention.

## Methods

### Study subjects

This study included RMPP patients (1–14 years old) who were diagnosed in the Pediatric Pulmonology Department, Shengjing Hospital of China Medical University from September 2016 to February 2020. The clinical, imaging and laboratory data of the included patients were retrospectively obtained from the hospital’s medical records. All participants had oral consent, and written informed consent was obtained from the parents of each participant in the study prior to enrollment. All patients had signs and symptoms of pneumonia on admission, including fever, cough, abnormal lung auscultation, and new infiltrates on chest radiographs. The diagnosis of MP pneumonia was based on the following three items: (1) Acute respiratory symptoms, including fever, sputum or wheezing; (2) Chest CT examination results suggested inflammatory infiltration, with/without abnormal lung sounds, such as wheezing or crackling; (3) Serological test MP-IgM was positive, and MP ribonucleic (RNA) test in pharyngeal swab or alveolar lavage fluid (BALF) was positive [14]. The diagnosis of RMPP is based on the presence of persistent fever and clinical and radiological deterioration after 7 days or more of appropriate macrolide therapy [15]. The diagnosis of BO was based on the observation of sub-segmental and sub-subsegmental bronchi distal occlusion under bronchoscopy, and imaging manifestations included atelectasis, bronchiectasis, and localized emphysema [8]. This study excluded patients with tuberculosis, asthma, repeated respiratory infections, bronchiectasis, or other chronic lung diseases; with a history of severe pneumonia before; with a history of congenital heart disease, chronic liver and kidney insufficiency, and immunodeficiency.

For the BO group, the following criteria needed to be met (in the group of RMPP combined with bronchitis obliterans, the following criteria needed to be met): (1) Children with RMPP were diagnosed; (2) Reexamination of chest X-ray or chest CT after 2–6 months from the acute phase showed the presence of inflammatory malabsorption, atelectasis, localized ventilation shadow and bronchiectasis; (3) The fiberoptic bronchoscopy was performed in our hospital, and tracheobronchial occlusion was found under the bronchoscopy. For the non-BO group, the following criteria were met: (1) Children with RMPP were diagnosed; (2) There was no bronchitis obliterans-related manifestation under bronchoscope; or (3) Pulmonary consolidation showed good recovery on lung CT within 2 months.

This study was approved by the Ethics Committee of Shengjing Hospital of China Medical University. Before enrollment, written informed consent was obtained from at least one guardian of each patient.

### Data collection

The clinical information was collected retrospectively from the patients’ medical records during acute infection, including age, gender, clinical symptoms and signs, intrapulmonary and extrapulmonary complications, fever duration and treatment. All patients underwent chest X-ray examination on admission, showing clear focal or segmental infiltration, with or without pleural effusion, and whether the consolidation area exceeded 2/3 of the lung lobes. Peripheral blood samples were collected on admission to determine complete blood count, albumin (ALB), C-reactive protein (CRP), PCT, lactate dehydrogenase (LDH), and T lymphocyte subsets. In addition to the MP-RNA test and MP-IgM test, other microbiological tests were carried out, including protein purification derivative tests, blood bacterial cultures, BALF bacterial cultures, and nasopharyngeal aspirates/swabs to detect common respiratory virus antigens (respiratory syncytial virus, influenza virus, adenovirus and parainfluenza virus), and serological testing of chlamydia pneumoniae. During the treatment, the timing and course of treatment of macrolides, the timing and course of glucocorticoid use, the timing of fiberoptic bronchoscopy, the changes observed by bronchoscopy, and whether there was plastic bronchitis were all recorded.

### Statistical analysis

The SPSS software (V23.0, IBM, New York, USA) and R software (V.3.3.3, R Foundation for Statistical Computing, Vienna, Austria) were adopted for statistical analysis. For categorical variables, χ^2^ or Fisher’s exact test was used. The skew distribution data was expressed as the median value (interquartile value), and the Mann–Whitney U rank sum test was used to compare the two groups. Logistic regression analysis was performed to determine the risk factors related to the occurrence of BO in RMPP patients. And a nomogram was constructed based on the results of previous multivariate analysis. The area under the receiver operating characteristic curve (AUC) and the Hosmer–Lemeshow goodness-of-fit test were used to evaluate the performance of the predictive model. A two-tailed p < 0.05 was considered statistically significant.

## Results

### Comparison of general information and clinical characteristics

A total of 141 patients with confirmed RMPP were included in this study. Among the 141 cases of RMPP patients, Outcome of BO occurred in 65 patients with RMPP. All patients have symptoms and signs of pneumonia, including fever, cough, and abnormal breath sounds on auscultation. The average time of BO found by bronchoscopy is 18 (24, 37) days after infection. There was no significant difference in gender and age at diagnosis between the non-BO group and the BO group (Table 1). However, the number of fever days in the BO group was significantly higher than that in the non-BO group (p < 0.05). There were 28 cases of mixed infection in the BO group, of which 10 cases were co-infected with influenza virus and 3 cases were co-infected with adenovirus. And there were 20 cases of mixed infection in the non-BO group, of which 4 cases were co-infected with the influenza virus. Compared with non-BO patients, patients with BO had a higher risk of mixed infection (p < 0.05), especially co-infection with influenza virus (p < 0.05). The children with RMPP were followed up after discharge. As the results showed, 48 patients (73.8%) in the BO group had symptoms such as chronic cough, recurrent pneumonia and wheezing; 16 patients (24.6%) had no obvious symptoms, and 1 patient (1.5%) was lost to follow-up. In the non-BO group, 11 cases (14.5%) developed chronic cough, recurrent pneumonia and wheezing; 63 cases (82.9%) had no obvious symptoms, and 2 cases (2.6%) were lost to follow-up.

**Table 1:** Comparison of clinical characteristics and follow-up

Clinical index	BO (n = 65)	Non-BO (n = 76)	Z/χ^2^	p
Clinical Characteristics				
Gender (male/female)	38/27	42/34	0.146	0.702
Age (years old)	6.0 (5.0,8.0)	6.0 (5.0,8.7)	− 0.382	0.703
Fever time (days)	14.0 (10.0,17.0)	10.5 (8.0,12.0)	− 4.296	< 0.001
Mixed infection (n %)	28 (43.1%)	20 (26.3%)	4.384	0.036
Bacteria (n %)	8 (12.3%)	2 (2.6%)	3.618	0.057
EB virus (n %)	10 (15.4%)	15 (19.7%)	0.455	0.500
Influenza virus (n %)	10 (15.4%)	4 (5.3%)	4.013	0.045
Adenovirus (n %)	3 (4.6%)	–		0.096*
Follow-up				
Chronic cough	34 (52.3%)	2 (2.6%)	42.897	< 0.001
Recurrent pneumonia	6 (9.23%)	28 (36.8%)	14.597	< 0.001
Recurrent wheezing	4 (6.2%)	3 (3.9%)	0.045	0.832
Asymptomatic	16 (24.6%)	63 (8.3%)	48.301	< 0.001

*p represents the p value calculated by Fisher’s exact probability method

The comparison of laboratory data found that there was no statistically significant difference in LDH and PCT levels between the two groups (Table 2). The WBC count, D-dimer and CD8 levels of the BO group were higher than those of the non-BO group, while the ALB level was significantly lower than that of the non-BO group (p < 0.05). Furthermore, there was a significant difference in the distribution of lung lobes between the BO group and the non-BO group (Table 3). The proportion of patients with lung infiltration areas larger than 2/3 of the lung lobes in the BO group was significantly higher than that in the non-BO group (p < 0.05). The proportion of patients in the BO group who received macrolides within 5 days of the disease course (38.5%) was significantly lower than that of the non-BO group (78.9%), while the total course of macrolides in the BO group was significantly higher than that of the non-BO group (p < 0.05, Table 4). Among them, the course of erythromycin treatment in the BO group was significantly higher than that in the non-BO group (p < 0.05). There was no significant difference in the proportion of patients receiving glucocorticoid therapy between the two groups (p > 0.05), but the proportion of patients in the BO group receiving glucocorticoid therapy within two weeks of the disease course (44.6%) was significantly lower than that of the non-BO group (81.6%) (p < 0.05). A total of 127 patients with RMPP completed fiberoptic bronchoscopy, including 65 cases (100%) in the BO group and 62 cases (81.6%) in the non-BO group (p < 0.05). In addition, the treatment time under fiberoptic bronchoscopy in the BO group was significantly later than that in the non-BO group (p < 0.05), and the proportion of patients receiving fiberoptic bronchoscopy within 2 weeks of the disease course was significantly lower than that in the non-BO group (p < 0.05). A total of 27 patients underwent twice bronchoscopy, 25 (38.5%) in the BO group and 2 (2.6%) in the non-BO group (p < 0.05). The proportion of bronchial phlegm plugs and plastic bronchitis found in the BO group during the first bronchoscopy in the acute phase was higher than that of the non-BO group (p < 0.05).

**Table 2:** Comparisons of Laboratory Findings

Laboratory data	BO (n = 65)	Non-BO (n = 76)	Z/χ^2^	P
WBC count(1 × 10^9^/L)	9.76 (7.24,13.52)	8.4 (6.24,10.46)	− 2.912	0.004
Neutrophil (%)	67.9 (58.55,76.15)	64.7 (60.15,72.42)	− 1.166	0.243
Lymphocytes (%)	20.3 (13.55,28.50)	22.5 (15.22,27.57)	− 0.606	0.545
CRP (mg/L)	23.8 (8.14,62.05)	24.7 (13.55,58.15)	− 0.422	0.673
LDH (U/L)	411.0 (306.00,559.00)	378.0 (307.25,453.75)	− 1.148	0.137
PCT (10 ng/mL)	0.15 (0.08,0.27)	0.15 (0.08,0.30)	− 0.381	0.703
25OH-Vitamin D	16.7 (9.56,26.05)	19.9 (13.05,29.96)	− 1.657	0.097
D-dimer (ng/mL)	832.0 (517.50,212.00)	603.0 (361.50,1343.50)	− 2.070	0.038
ALT (U/L)	25.0 (15.50,52.00)	20.5 (13.25,41.75)	− 1.246	0.213
ALB (g/L)	33.8 (30.85,36.75)	37.7 (34.30,39.35)	− 4.238	< 0.001
Serum NK (%)	10.0 (6.00,17.75)	11.0 (7.52,14.65)	− 0.842	0.400
Absolute value of NK	146.0 (74.50,338.50)	170.5 (80.00,297.75)	− 0.041	0.967
CD4 + (%)	36.6 (28.60,35.40)	32.3 (30.40,40.75)	− 0.190	0.849
CD8 + (%)	30.3 (26.30,35.40)	27.6 (23.20,32.57)	− 2.381	0.017
CD4/CD8	1.17 (0.92,1.44)	1.26(0.99,1.71)	− 1.328	0.184
NK in BALF (%)	8.0 (5.60,11.90)	8.95 (5.80,13.62)	− 1.204	0.229
CD4 in BALF (%)	27.5 (19.60,32.25)	34.95 (23.00,43.90)	− 1.950	0.051
CD8 in BALF (%)	46.0 (36.30,59.00)	42.1 (32.52,52.57)	− 1.597	0.110
Proportion of lobulated nuclear cells in BALF	45.0 (31.500,59.00)	37.5 (18.25,57.75)	− 1.940	0.052

*WBC* white blood cell; *CRP* C-reactive protein; *LDH* lactate dehydrogenase; *PCT* procalcitonin; *ALB* albumin; *ALT* alanine aminotransferase; *NK* Natural killer cells; *BALF* bronchoalveolar lavage fluid

**Table 3:** Comparisons of radiologic features

Factors	BO (n = 65)	Non-BO (n = 76)	Z/χ^2^	p
Extrapulmonary involvement (n %)	29 (44.6%)	31 (40.8%)	0.210	0.647
The consolidation range exceeding 2/3 of the lung lobes (n %)	44 (67.7%)	37 (48.7%)	5.178	0.023
Pleural effusion (n %)	27 (41.5%)	28 (36.8%)	0.325	0.569

*p represents the p value calculated by Fisher’s exact probability method

**Table 4:** Comparisons of treatment

Factors	BO (n = 65)	Non-BO (n = 76)	Z/χ^2^	p
Timing of macrolides < 5 days of disease course (n %)	25 (38.5%)	60 (78.9%)	22.985	< 0.001
Total course of macrolides (days)	12.0 (8.0,15.0)	10.0 (8.0,12.0)	− 2.186	0.029
Course of Azithromycin (days)	8.0 (8.0,10.0)	8.0 (8.0,8.0)	− 0.284	0.776
Course of Erythromycin (days)	3.0 (0.0,7.0)	1.5 (0.0,4.0)	− 2.171	0.030
Glucocorticoid	58 (89.2%)	65 (85.5%)	0.432	0.511
Timing of glucocorticoid < 2 weeks (n %)	29 (44.6%)	62 (81.6%)	20.916	< 0.001
Course of Glucocorticoid	5.0 (3.0,8.0)	2 (3.0,8.0)	1.907	0.057
Fiberoptic bronchoscopy (n %)	65 (100%)	62 (81.6%)	13.294	< 0.001
Treatment timing of fiberoptic bronchoscopy < 2 weeks (n %)	16 (24.6%)	51 (67.1%)	25.364	< 0.001
First treatment time of fiberoptic bronchoscopy (days)	17.0 (14.0,23.0)	12 (10.0,13.0)	− 6.379	< 0.001
Twice bronchoscopy	25 (38.5%)	2 (2.6%)	26.783	< 0.001
Phlegm plug (n %)	36 (55.4%)	29 (38.2%)	4.184	0.041
Mucosal necrosis (n %)	13 (20.0%)	7 (9.2%)	3.351	0.067
Plastic bronchitis (n %)	17 (26.2%)	6 (7.9%)	8.556	0.003

*p represents the p value calculated by Fisher’s exact probability method

### Multivariate regression analysis of the occurrence of BO in patients due to RMPP

The above possible influencing factors were used as independent variables, and whether BO occurred was used as dependent variables, then they were included in the multivariate analysis (Table 5). The analysis results showed that WBC count, ALB level, consolidation range more than 2/3 of the lung lobes, the use of macrolides within 5 days of disease course, the use of glucocorticoid or fiber bronchoscopy treatment within 2 weeks of disease course, and plastic bronchitis were independent factors influencing the occurrence of BO in patients due to RMPP.

**Table 5:** Results of the Multivariate Logistic Regression Analysis

Factors	B	S.E	Wald	p	OR	95% CI
WBC	0.136	0.066	4.193	0.041	1.145	1.006–1.304
ALB	− 0.126	0.063	3.995	0.046	0.881	0.779–0.998
The consolidation range exceeding 2/3 of the lung lobes	1.354	0.514	6.947	0.008	3.872	1.415–10.594
Timing of macrolides < 5 days of disease course	− 1.222	0.502	5.927	0.015	0.295	0.110–0.788
Timing of glucocorticoid < 2 weeks of disease course	− 1.734	0.568	9.338	0.002	0.176	0.058–0.537
Treatment timing of fiberoptic bronchoscopy < 2 weeks	− 1.498	0.525	8.147	0.004	0.224	0.080–0.625
Plastic bronchitis	2.444	0.730	11.210	0.001	11.516	2.754–48.152
Constant	4.473	2.499	3.203	0.074	87.604	

*WBC* white blood cell count; *ALB* albumin; *OR* odds ratio; *CI* confidence interval

### The nomogram of BO occurrence and the performance evaluation of the nomogram

**Fig. 1:**
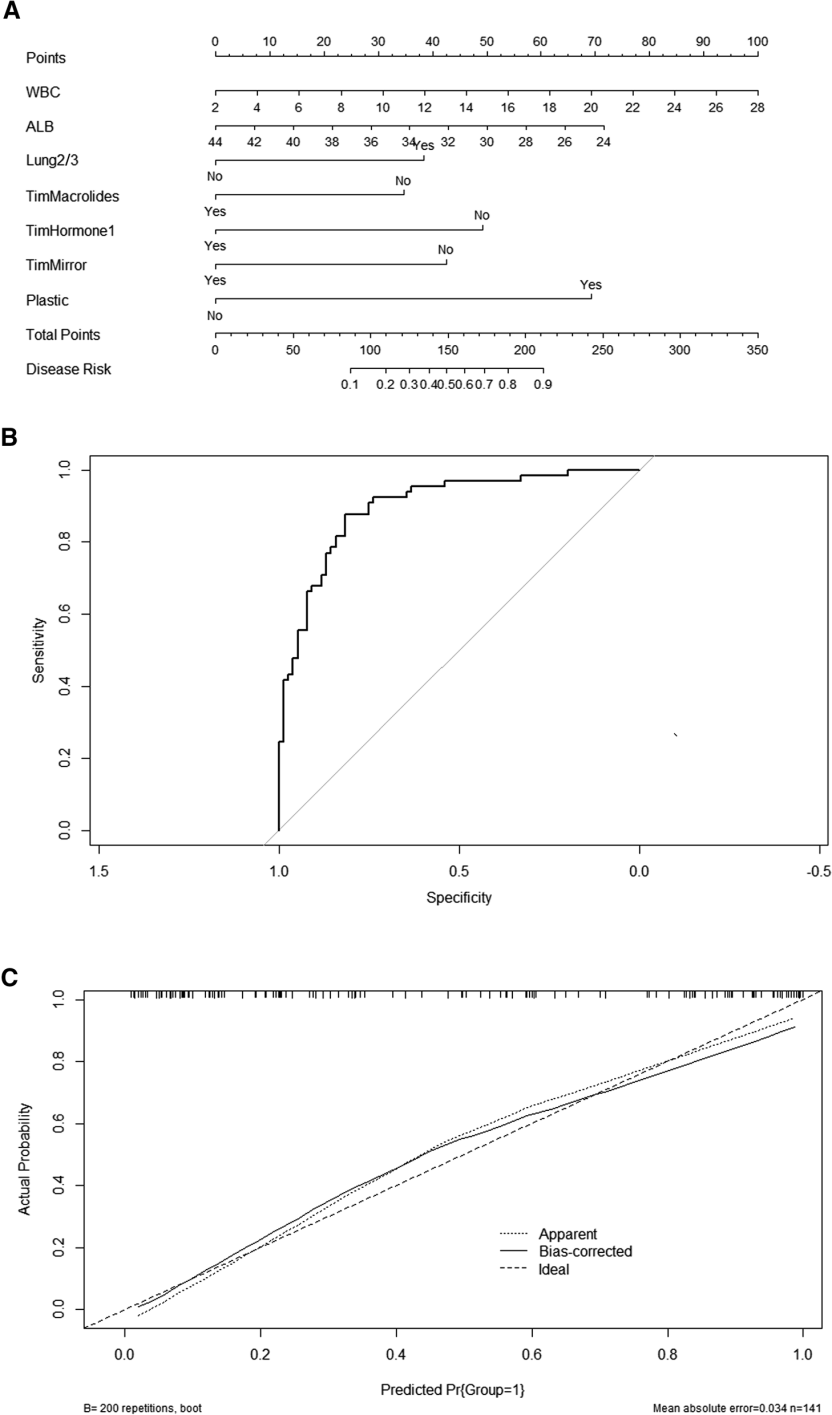
**A** The nomogram for calculating the risk score and predicting the risk of BO in RMPP patients. **B** Receiver operating characteristic (ROC) curve analysis in the main cohort. **C** Calibration curve analysis in the main cohort. The horizontal axis indicates the risk of BO occurrence predicted by the nomogram, and the vertical axis represents the actual observed risk of BO occurrence. *WBC* white blood cell count; *ALB* albumin; *Lung2/3* the consolidation range exceeding 2/3 of the lung lobes; *TimMacrolides* timing of macrolides < 5 days of disease course; *TimHormone1* timing of glucocorticoid < 2 weeks of disease course; *TimMirror* treatment timing of fiberoptic bronchoscopy < 2 weeks of disease course; *Plastic* plastic bronchitis

The seven risk factors obtained from logistical regression analysis established a nomogram of the risk of BO (Fig. 1A). The nomogram was generated by assigning a weighted score to each independent influencing factor. The highest score is 160 points, and the range of BO incidence is 0.1 to 0.9. A higher score calculated from the sum of the distribution points of each high-risk factor in the nomogram corresponds to a higher risk of occurrence. The Hosmer–Lemshaw test was adopted for a model test, and the test result was p = 0.692, R^2^ = 0.586, indicating that the information in the current data had been fully extracted. The AUC shows that the predictive power of the predictive model in the main cohort is 0.899 (95% CI 0.848–0.950) (Fig. 1B). The calibration chart shows that the nomogram has a sufficient degree of fit for predicting the incidence of BO in RMPP patients (Fig. 1C).

## Discussion

Previous studies have used multivariate regression analysis to determine the risk of extrapulmonary complications in MPP patients, such as pulmonary necrosis [16] and intrabronchial mucus embolism [17]. However, studies on the risk of BO coused by RMPP patients have not been reported yet. In the present study, we determined seven factors related to the risk of BO in children caused by RMPP through single factor and multivariate regression analysis, including WBC count, ALB level, consolidation range exceeding 2/3 of the lung lobes, the use of macrolides within 5 days, the use of glucocorticoid or fiber bronchoscopy within 2 weeks of disease course, and plastic bronchitis, all of which were used to establish a nomogram of the risk of BO. As far as we know, this is the first nomogram study of the risk of BO caused by RMPP. Based on AUC and calibration curve evaluation, this novel nomogram showed satisfactory performance in the research cohort. Therefore, the nomogram can be effectively used in clinical practice, which helps predict the development of BO in children due to RMPP, and emphasizes the importance of the timing of treatment with macrolides, glucocorticoids, and bronchoscopy after MPP.

Previous studies have shown that there is no difference in clinical characteristics and disease severity between MPP patients co-infected with viral and/or bacterial pathogens and MPP patients without any respiratory virus co-infection [18]. However, Lee et al. [19] have found that respiratory virus co-infection is a risk factor for BO after infection in children with MPP. In the current study, we found that although the proportion of children with RMPP pneumonia complicated with influenza infection was higher than that of non-BO children, multivariate regression analysis found that influenza infection was not a risk factor for the development of BO after RMPP. It is widely known that adenovirus infection is one of the risk factors for the development of BO in children [20]. In the current study, we did not find an association between adenovirus co-infection and the development of BO after RMPP, which may be related to the small sample size.

Inflammatory mediators are involved in the immune pathogenesis of MP infection [21]. As a negative acute-phase protein synthesized by the liver, serum ALB can rapidly decrease in acute infection [22]. The reduction of serum albumin level is considered to be a biomarker of local and systemic inflammation, and has important clinical value in predicting the severity and prognosis of pneumonia [22–24]. As a specific marker of fibrinolysis, D-dimer reflects the ability to dissolve fibrin [25]. Studies have shown that the clinical symptoms and chest imaging findings of MPP patients with elevated D-dimer levels are more severe than those with normal D-dimer levels [26, 27]. The increase of serum D-dimer level in patients with RMPP suggests excessive inflammation and prolonged vascular endothelial injury [28]. In the current study, compared with the non-BO group, the WBC count, CD8+ ratio and serum D-dimer level of the BO group were significantly increased, while the serum ALB level was significantly reduced. In addition, high WBC counts and low ALB level are independent risk factors for the occurrence of BO, suggesting that patients with RMPP complicated by BO have stronger systemic inflammatory responses. Persistent MP antigen stimulation and/or invasion greatly increases the occurrence of severe lung disease and pulmonary and extrapulmonary complications [29]. In the present study, the severity of pneumonia is an independent risk factor for the development of BO after RMPP, which suggests the need to monitor the lesions of RMPP patients in time.

Macrolides are the first choice of drug for MP infection in children [30]. Some research shows many MP isolates in clinical samples showed resistance to macrolides [31]. Macrolide resistance in MP may weaken the response to drug therapy [32–34]. The presence of macrolide-resistant MP has been reported to be mainly related to persistent clinical symptoms such as fever, prolonged hospital stay, and increased antibiotic replacement rate [35]. Therefore, macrolide resistance may be one cause of RMPP [36]. However, some studies have shown that there is no significant difference in the detection rate of drug resistance genes between RMPP patients and children with ordinary MPP [37], revealing that macrolide resistance may not be the main cause of RMPP [38]. Although many MP isolates in clinical samples showed resistance to macrolide drugs in vitro, due to the differences in the pharmacokinetics, pharmacodynamics and tissue concentrations of the drugs in individuals, cultivated drug resistance in vitro does not mean that drug resistance will appear in vivo, and its clinical significance needs further evaluation. In the course of treatment, clinical symptoms and response to treatment are still used to evaluate the effectiveness of antibiotics and whether drug treatment needs to be replaced. As for patients who are weaken to macrolide therapy and who is considered as macrolide drug-resistant patients, antibiotics can be replaced with tetracyclines and quinolones. However, tetracycline drugs are because of the possible side effects such as yellowing of teeth or enamel dysplasia, it is not recommended for children patients under 8 years of age. Besides, quinolone antibiotics may have an adverse effect on bone development, and its safety for children has not been established. Therefore, it is contraindicated with children under 18 years of age. This is a retrospective study and is affected by the following factors: there was no necessary connection between in vitro test results and in vivo treatment effects; MP culture conditions were strict and time-consuming; drug resistance gene testing has a higher economic cost for patients; in this study, according to statistics, the age group of RMPP patients was mostly 5–8 years old. Even if drug-resistant MP exists, most children patients cannot be recommended to use tetracyclines and quinolones for treatment; therefore, this study did not conduct macrolide drug-resistant MP gene detection. It is worth discussing whether changing antibiotic therapy after MP resistance can benefit patients. We will further study this issue in the future.

Inflammatory mediators participate in the immune pathogenesis of *Mycoplasma pneumoniae* infection [5]. Moreover, the anti-inflammatory and immunosuppressive effects of macrolides have been widely confirmed. In short, macrolides exert their effects by reducing the accumulation of neutrophils in the epithelium of airway, resulting in a decrease of the local production of inflammation mediator (IL-8 pro-IL-1β and TNF-α, etc.) [39, 40]. In the current study, we found that compared with the BO group (38.5%), the non-BO group (78.9%) used a higher proportion of macrolides within 5 days of the disease course. While the levels of inflammatory mediators (such as WBC count, D-dimer level) of non-BOS patients are significantly lower than those of BO patients. Therefore, we speculated that the anti-inflammatory and immunosuppressive effects of macrolides may be an important reason for reducing the occurrence of BO in patients with RMPP. We found that patients with BO showed longer treatment time for macrolides and glucocorticoids. This may be due to the late treatment time leading to excessive inflammation caused by infection, leading to prolonged clinical course. Thus, we suggest that the use of macrolides as soon as possible can reduce the occurrence of BO.

The pathogenesis of RMPP is still currently believed to be related to the hypersecretion of airway mucus [41], hypercoagulable state [28], mixed with bacterial or viral infection [42] and excessive immune response caused by toxin production of community-acquired respiratory distress syndrome [43]. In addition, bronchoscopic alveolar lavage to remove airway phlegm and inflammatory factors in time [39], and glucocorticoid therapy to suppress excessive immune response are also important means to treat RMPP [40, 44]. Moreover, this study also found that plastic bronchitis caused by airway mucus hypersecretion was a risk factor for BO. The use of fiberoptic bronchoscopy and hormone therapy within 2 weeks were protective factors for BO which was caused by RMPP. For RMPP patients with poor effect in the treatment of antibiotics and corticosteroids, fiberoptic bronchoscopy within 2 weeks of the disease course may effectively prevent the occurrence of BO.

Plastic bronchitis is a rare and underdiagnosed disease [45]. Influenza virus and *Mycoplasma pneumoniae* infection are usually common causes of plastic bronchitis [46]. Plastic casts are caused by a variety of inflammatory cell infiltration and inflammatory mediators, leading to congestion, edema, necrosis, and lumen obstruction of the tracheal mucosa [47]. In the current study, we found through fiberoptic bronchoscopy that bronchial phlegm plugs and plastic bronchitis found in the BO group during the first bronchoscopy in the acute phase was higher than that of the non-BO group, which may be related to the excessive inflammatory reaction and bronchial endometrial damage in RMPP leads to the occurrence of BO. Moreover, the occurrence of plastic bronchitis was an independent risk factor for the occurrence of BO in RMPP patients, suggesting that the plastic under fiberoptic bronchoscopy may indicate the occurrence of BO.

The nomogram is very useful in using individual variables to predict the probability of a clinical event occurring in an individual. As an alternative, unlike multivariate regression analysis, a nomogram is a graphical description of a statistical model that calculates the probability of an individual patient’s specific outcome with satisfactory accuracy [48]. The nomogram has its own limitations. For example, when the covariate measurement changes, the performance of the nomogram may change greatly, and the influence of the nomogram-assisted decision-making on patient outcomes still needs to be studied [49]. Nonetheless, nomograms have been widely used to predict the short-term and long-term outcomes of various diseases (such as cancer and diabetes) [50]. In the current research, we provided a simple and easy-to-use risk prediction nomogram for the first time, which contains five factors that affect the occurrence of BO. The satisfactory performance of this model is reflected in its high predictive ability, and its AUC for the study cohort is greater than 0.8. The nomogram may improve and provide new ideas for the early identification and intervention of BO in RMPP patients.

There are still some limitations in this study. First of all, the study population was small, and only patients from a single center were included. Secondly, this is a retrospective study based on reviewing medical records, and patients with incomplete medical records were excluded. Thirdly, considering that macrolide resistance may be related to the occurrence of RMPP, antimicrobial susceptibility tests are still needed to classify patients with BO. In addition, multiple samples are still required to be listed to verify the feasibility of the nomogram model in this study. In order to further confirm the results of this study, it is necessary to conduct a multi-center in-sample study.

## Conclusions

In summary, our research has developed a nomogram with seven factors, including WBC count, ALB level, consolidation range exceeding 2/3 of the lung lobe, the use of macrolides within 5 days of disease course, the use of glucocorticoid within 2 weeks of disease course, the treatment time fiberoptic bronchoscopy within 2 weeks of disease course and plastic bronchitis, with the purpose to predict the risk of BO in children due to RMPP. The nomogram has performed well and may help the clinical identification and decision-making of BO patients caused by RMPP.

## Data Availability

The data that support the findings of this study are available from the corresponding author upon reasonable request.

## Abbreviations

BO: Bronchitis obliterans
RMPP: Refractory *Mycoplasma pneumoniae* pneumonia
MP: Mycoplasma pneumonia
CAP: Community-acquired pneumonia
MPP: *Mycoplasma pneumoniae* Pneumonia
NP: Necrotizing pneumonia
RNA: Ribonucleic acid
WBC: White blood cell count
ALB: Albumin
CRP: C-reactive protein
LDH: Lactate dehydrogenase
AUC-ROC: Area under the receiver operating characteristic curve
CI: Confidence interval
